# GC/EI-MS method for the determination of phytosterols in vegetable oils

**DOI:** 10.1007/s00216-021-03730-9

**Published:** 2021-10-30

**Authors:** Sarah Schlag, Yining Huang, Walter Vetter

**Affiliations:** Institute of Food Chemistry (170B), University of Hohenheim, Garbenstraße 28, 70599 Stuttgart, Germany

**Keywords:** Vegetable oil, Phytosterol, Triterpenol, Sterol composition, Gas chromatography with mass spectrometry (GC/MS), Fatty acid pyrrolidide

## Abstract

Sterols are a highly complex group of lipophilic compounds present in the unsaponifiable matter of virtually all living organisms. In this study, we developed a novel gas chromatography with mass spectrometry selected ion monitoring (GC/MS-SIM) method for the comprehensive analysis of sterols after saponification and silylation. A new referencing system was introduced by means of a series of saturated fatty acid pyrrolidides (FAPs) as internal standards. Linked with retention time locking (RTL), the resulting FAP retention indices (RI_FAP_) of the sterols could be determined with high precision. The GC/MS-SIM method was based on the parallel measurement of 17 SIM ions in four time windows. This set included eight molecular ions and seven diagnostic fragment ions of silylated sterols as well as two abundant ions of FAPs. Altogether, twenty molecular ions of C_27_- to C_31_-sterols with 0–3 double bonds were included in the final method. Screening of four common vegetable oils (sunflower oil, hemp oil, rapeseed oil, and corn oil) enabled the detection of 30 different sterols and triterpenes most of which could be identified.

**Graphical abstract:**

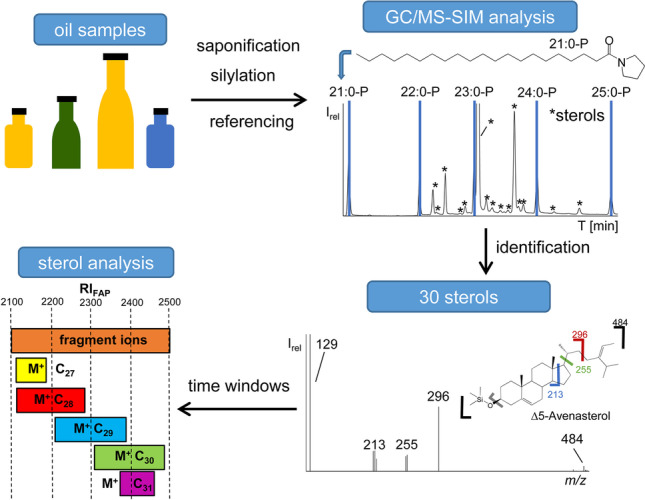

**Supplementary Information:**

The online version contains supplementary material available at 10.1007/s00216-021-03730-9.

## Introduction

Phytosterols make up a substantial part of the unsaponifiable matter of vegetable oils and fats [1]. These bioactive compounds are considered healthy components of our diet [2]. The only known possible adverse effect has been observed in people suffering from phytosterolaemia which is a very rare inherited lipid storage disease [3]. The most relevant positive effect is reduction of the uptake of cholesterol in humans and thus lower serum cholesterol levels. Specifically, daily doses of 1–2 g of 4,4-desmethylsterols or stanols may reduce the serum cholesterol level by about 10% [4, 5]. Since very high phytosterol levels may reduce the blood level of β-carotene, this health claim was limited to a maximum intake of 3 g/day phytosterols [6]. Further proposed beneficial health benefits of phytosterols are antioxidative and anti-inflammatory effects as well as anticarcinogenic properties [7]. The average daily consumption of β-sitosterol was shown to be sufficient for an improvement of the clinical symptoms of benign prostate hyperplasia [7, 8]. 4,4-Dimethylsterols (e.g., cycloartenol) and pentacyclic amyrins are currently part of investigations, as they also show beneficial health effects, but by now, they were only tested in medical doses [9].

**Fig. 1:**
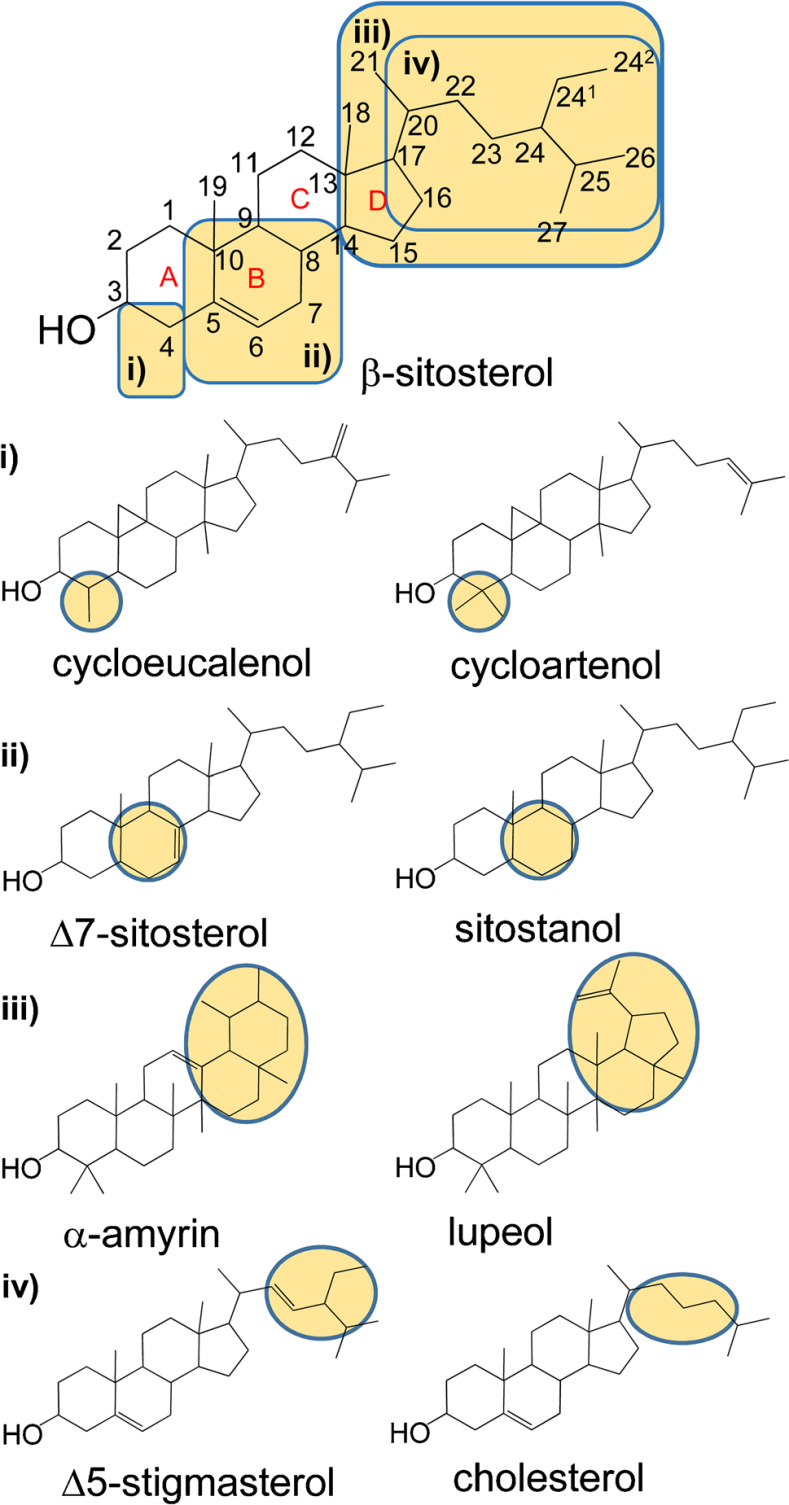
Structures of important sterols and pentacyclic triterpenes. Sterols can differ in the substitution at C4 (i), in the saturation of the B-ring (ii), and in the substitution of the side chain (iv). Amyrins (iii) have a similar structure

Chemically, sterols belong to the family of triterpene alcohols and are characterized by a 1,2-cyclophenanthrene backbone which is generally substituted with both a hydroxyl group on C-3 and a branched alkyl chain on C-17. The ring system (and the alkyl chain) of sterols can either be saturated (subgroup of stanols) or carry one or more double bonds (DBs), i.e., the subgroup also named sterols. DBs are often present at C-5 (designated as ∆5-sterol) and/or C-7 (designated as ∆7- or ∆5,7-sterol) (Fig. 1ii). In addition, the branched alkyl chain can also feature one or, scarcely, two DBs at different positions and may vary in substitution pattern and stereochemistry (Fig. 1iv) [10, 11]. Sterols can further be classified according to the degree of substitution at C-4, i.e., 4-desmethylsterols; 4-methylsterols; and 4,4-dimethylsterols, respectively (Fig. 1i). 4,4-Dimethyl- and 4-methylsterols serve as precursors of 4-desmethylsterols in sterol biosynthesis and typically represent only a small amount of the total sterol content of food, but exceptions are known (e.g., tomato seed oil, ~ 40%) [10, 12]. Other triterpene alcohols such as the pentacyclic amyrins and lupeol (Fig. 1iii), which are often determined together with sterols, are found as well in food [13–15].

Altogether, more than 250 structural variants were described in the scientific literature, but only ~ 15 or less sterols are currently determined in routine analysis of vegetable oils [16]. This is mainly due to (i) the wide concentration range of individual sterols, (ii) the lack of reference standards, and the last but not least (iii) insufficiently sensitive and specific analysis methods. After enrichment of the non-saponifiable matter, sterols are mostly analyzed after silylation by means of gas chromatography with flame ionization detection (GC/FID) or mass spectrometry (GC/MS) [17–19]. The resulting gas chromatograms of sample extracts frequently feature peaks which cannot be assigned and thus remain unlabeled. In this way, however, valuable basic information is lost.

To overcome these problems, we aimed to develop a referencing system according to the concept of retention indices, which was initially introduced by Kováts for isothermal determination using logarithmic retention times [20] and was later adapted by van den Dool and Kratz [21] for the usage of a linear temperature-programmed method. Previous experience with fatty acid pyrrolidides (FAPs) used for structural analysis of fatty acids [22, 23] indicated that a group of homologues shares the retention time range of silylated sterols. Moreover, the concept was combined with the retention time locking (RTL) technique [24] and expanded by developing a GC/MS method operated in the selected ion monitoring (SIM) mode for the thorough analysis of silylated sterols. The method was applied to and tested with four typical edible oils.

## Materials and methods

### Samples

Four vegetable oils (sunflower oil, corn oil, hemp oil, and rapeseed oil) were randomly purchased at local supermarkets. They were stored at 4 °C in the absence of light until sample preparation.

### Chemicals and standards

Nitrogen (99.95% purity) and helium (99.999% purity) were from Westfalen Company (Münster, Germany). Ethanol (technical grade, distilled prior to use) was from BASF (Ludwigshafen, Germany), *n*-hexane (HPLC grade) was from Th. Geyer (Renningen, Germany), whereas potassium hydroxide (> 85%) and sodium sulfate (> 99%) were from Carl Roth (Karlsruhe, Germany). Stearic acid (18:0; 99%), lignoceric acid (24:0; > 99%), cerotic acid (26:0; ≥ 90%), and pyridine were from Sigma-Aldrich (Steinheim, Germany). Pyrrolidine (> 98%), heneicosanoic acid (21:0, ≥ 98%), tricosanoic acid (23:0, ≥ 95%), and pentacosanoic acid (25:0, ≥ 95%) were from TCI (Zwijndrecht, Belgium). Nonadecanoic acid (19:0, ≥ 99%), arachidic acid (20:0, ≥ 99%), and behenic acid (22:0, ≥ 97%) were from Fluka (Buchs, Switzerland). A semipure β-sitosterol standard (total sterols, 95%; unsaturated sterols calculated as β-sitosterol, 85%) was from Merck (Darmstadt, Germany). 5α-Cholestane (98%) was from Acros Organics (Geel, Belgium), and the silylating agent consisting of *N*,*O*-bis(trimethylsilyl)-trifluoroacetamide (BSTFA) and trimethylchlorosilane (TMCS), 99:1, v/v (SILYL-991), was from Macherey–Nagel (Düren, Germany).

### Preparation of fatty acid pyrrolidides via fatty acid trimethylsilyl esters

Pyrrolidides were prepared according to Vetter and Walther [23], with slight modifications. About 10 mg of a saturated fatty acid (i.e., 18:0, 19:0, 20:0, 21:0, 22:0, 23:0, 24.0, 25:0, and 26:0) was accurately weighed into a 4-mL screw cap vial. After slowly adding 1 mL of SILYL-991 solution and 1 mL pyrrolidine, the vials were closed and stored at room temperature for 3 days. Afterwards, the derivatization agent was evaporated under a gentle stream of nitrogen in a heating block kept at 50 °C. The dry residue was dissolved in 4 mL *n*-hexane. After adding 4 mL water, the tube was sealed and shaken and the organic phase was transferred into another tube. The procedure was repeated twice, and the organic phase was then dried with sodium sulfate and filtered through a funnel. The solvent was evaporated as described above, and the residue was re-dissolved in 1 mL *n*-hexane. For the preparation of the internal standard (IS) solution, aliquots of the individual FAP solutions (18:0-P to 26:0-P) and a solution of 5α-cholestane (conc.) were pipetted in one volumetric flask and diluted with *n*-hexane (FAP-IS solution). For the GC/MS analysis, the concentrations of the individual FAPs in the FAP-IS solution were adjusted to a point which allowed detecting them in full scan mode by means of the extracted fragment ion *m/z* 113. This corresponded to approximately 3 ng (3 μg/mL) for 18:0-P to 25:0-P, 10 ng (10 μg/mL) for 26:0-P considering the weight of the synthesis product. Similarly, 5α-cholestane was adjusted to 6 μg/mL.

### Sample saponification

Saponification was performed according to Hammann and Vetter [25]. In short, vegetable oil (~ 20 mg) was weighed into a 6-mL test tube and 1.8 mL of ethanol and 0.2 mL of KOH in water (50%, w/w) were added. The tube was sealed and heated for 1 h to 80 °C. After cooling to room temperature with an ice bath, 0.5 mL of water was added and the unsaponifiable matter was extracted with 2 mL of *n*-hexane.

### Trimethylsilylation of the unsaponifiable matter

Since the amount of the extracted unsaponifiable matter was too small to be precisely weighed, the amount was estimated on the premise that the unsaponifiable matter in a vegetable oil contributed ~ 2% to the lipid extract. Accordingly, 50 μL aliquots of the solutions of the unsaponifiable matter were pipetted into 1.5-mL screw cap vials equipped with a 200-μL insert and the solvent was evaporated to dryness at 40 °C under a gentle stream of nitrogen. According to Hammann and Vetter [25], SILYL-991 solution (50 μL) and distilled pyridine (25 μL) were added and the closed vials were heated to 60 °C for 30 min. Thereafter, the solvent was evaporated as described above and the residue was re-dissolved in 100 μL of the FAP-IS solution. This solution was used for the GC/MS analysis.

### Gas chromatography coupled to mass spectrometry

The GC/MS analysis was performed on a 6890/5973 N GC/MS system (Hewlett-Packard/Agilent, Waldbronn, Germany). Splitless injections (1 μL) were carried out via an MPS 2 autosampler (Gerstel, Mülheim, Germany) onto a 2 m, 0.25 mm i.d. Zebron guard column with deactivated tubing (Phenomenex, Aschaffenburg, Germany). The guard column was linked via a deactivated press fit connector (BGB Analytik, Rheinfelden, Germany) to a 30 m, 0.25 mm i.d. analytical column coated with a 0.25-μm film consisting of 95% methyl polysiloxane and 5% phenyl polysiloxane (Optima 5 HT, Macherey–Nagel, Düren, Germany). The carrier gas helium (purity 99.999%) was used at a flow rate of 1.0 mL/min. The column oven was programmed as follows: After 1 min at 55 °C, T was raised at 20 °C/min to 255 °C, then at 1.5 °C/min to 283 °C, and finally at 15 °C/min to 300 °C (hold time, 11 min). Injector, transfer line, ion source, and quadrupole temperatures were set at 250 °C, 280 °C, 230 °C, and 150 °C, respectively. The full scan mode covered *m/z* 50–650 after a solvent delay of 18 min. As a starting point, two different GC/MS-SIM methods were used (Table 1). Sterol identification was based on relative retention time (RRT), molecular ion, and characteristic fragment ions. The corresponding ^13^C isotope peak ([M-14]^+^) of the [M-15]^+^ fragment ions was compared to a known sterol to exclude a possible molecular ion of a co-eluting unknown sterol.

**Table 1:** Data of the GC/MS-SIM methods for silylated sterols and triterpene alcohols and the internal fatty acid pyrrolidide (FAP) standards initially used during method development

	Time window	Number of carbon atoms	Molecular ions				Fragment ions
			0	1	2	3	
SIM 1	20–41.8 min	28	474.4	472.4	470.4	468.4	113.0*; 126.0*; 129.1; 211.2; 213.2; 215.2; 253.2; 255.2; 296.2
		29	488.4	486.4	484.4	482.4	
SIM 2	20–29.6 min	27	460.4	458.4	456.4	454.4	
		30	502.5	500.4	498.4	496.4	
	29.6–32.5 min	30	502.5	500.4	498.4	496.4	113.0*; 129.1; 189.2; 211.2; 213.2; 215.2; 218.3; 253.2; 255.2; 296.2
		31	516.5	514.5	512.5	-	
	32.5–41.8 min	30	502.5	500.4	498.4	496.4	113.0*; 126.0*; 129.1; 211.2; 213.2; 215.2; 253.2; 255.2; 296.2
		31	516.5	514.5	512.5	510.5	

^*^Fragment ions used for FAPs, all other for silylated sterols and triterpene alcohols

Retention times were locked by means of a five-point calibration. Namely, the FAP-IS solution was measured with the nominal method pressure and with pressures deviating at − 20%, − 10%, + 10%, and + 20% from the nominal method pressure. The retention time of 24:0-P was set at 32.972 min. The method was relocked every second week and after each cleaning step of the instrument.

### Calculation of FAP Indices According to the Kováts Approach

The FAP retention index (RI_FAP_) of silylated sterols was calculated for a temperature-programmed method in relation to the FAPs in the FAP-IS solution [21] (Eq. 1):

1 $${\mathrm{RI}}_{\mathrm{FAP}}=100\bullet \left[n+\left(\frac{t\left(\mathrm{silylated}\ {sterol}\right)-t(n)}{t\left(N\right)-t(n)}\right)\right]$$

with “*n*” and “*N*” being the number of carbon atoms of the FAP eluting before and after the silylated sterol and “*t*” being the corresponding retention times. Four digit numbers were assigned, whereof the first two represented the FAP eluting before the silylated sterol and the last two indicating the relative position according to Eq. 1.

## Results and discussion

### Synthesis of pyrrolidides of saturated fatty acids

**Fig. 2:**
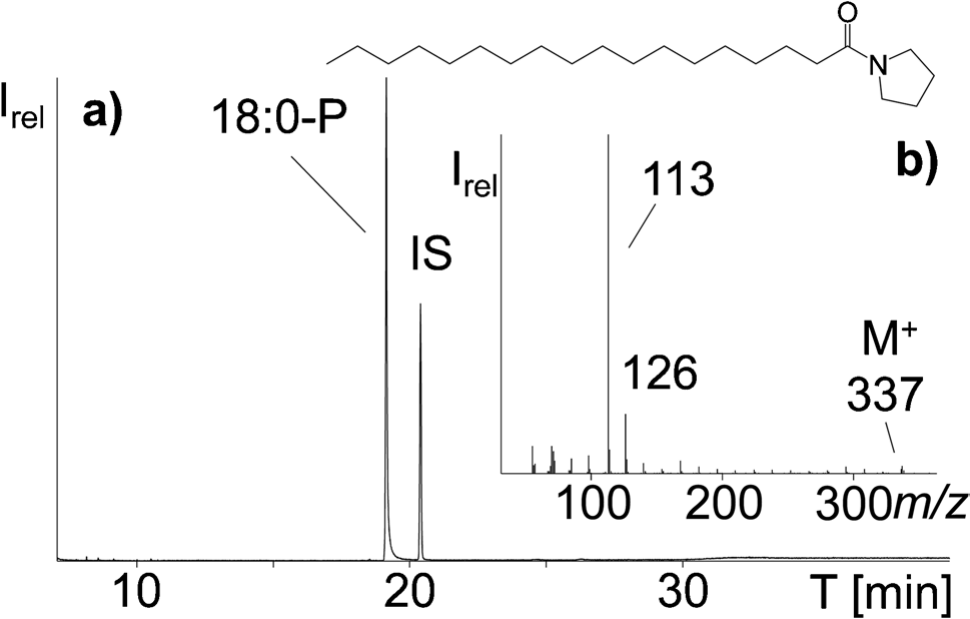
(a) GC/MS-full scan chromatogram of the synthesized 18:0-P (together with the internal standard (IS, 5α-cholestane)) and (b) GC/MS-full scan mass spectrum of 18:0-P

**Fig. 3:**
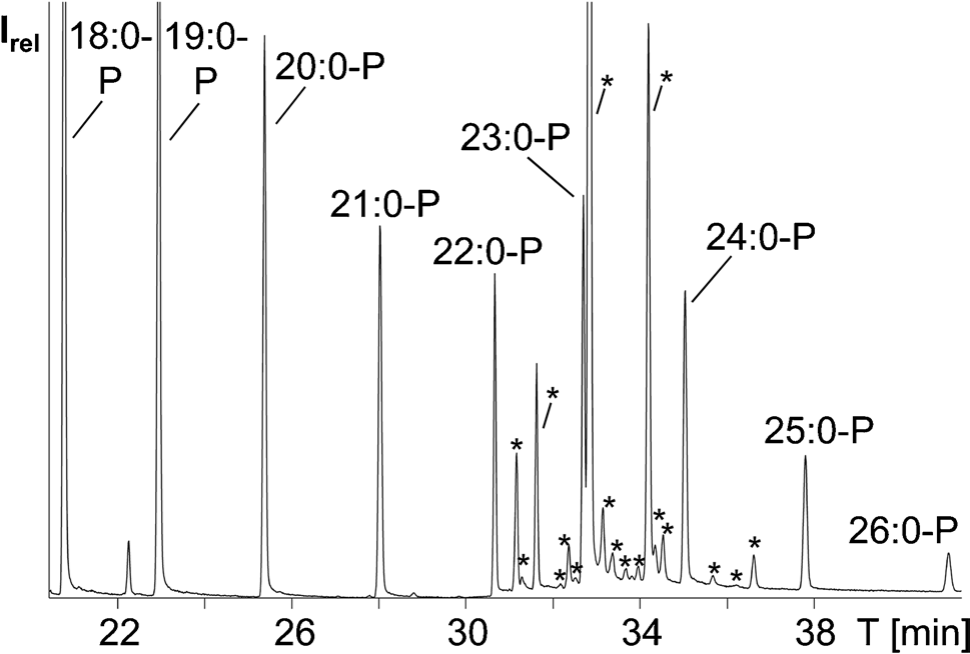
GC/MS-SIM chromatogram of the silylated unsaponifiable matter of sunflower oil with synthesized fatty acid pyrrolidides (18:0-P to 26:0-P) as internal standard. Sterols are marked with an asterisk

Adaption and upscaling of the method of Vetter and Walther (see experimental part) resulted in good yields of highly pure FAPs (Fig. 2a) except for the yield of 26:0-P due to its very poor solubility in *n*-hexane. Also, 26:0-P produced a much smaller peak in the GC/MS chromatogram compared to the other FAPs. Synthesized FAPs were not weighed but aliquots were diluted to approximately 50 μg/mL based on the used weight of the fatty acids. Final volumes for combination were then selected to give approximately comparable peak intensities except 26:0-P which generated a smaller peak (Fig. 3).

As anticipated, FAPs eluted in the retention time range of silylated sterols (i.e., between 21:0-P and 25:0-P, Fig. 3). Hence, the GC/MS chromatogram was divided into four sections, whereas conventional IS in sterol analysis like 5α-cholestane and betulin eluted either before or after all sterols [16, 26]. Initial screening of various samples indicated that 18:0-P to 20:0-P and 26:0-P were outside of the elution range of silylated sterols and thus not required in this study. For this reason, 26:0-P was not included in the final FAP-mix used in the following. However, the RRT of 26:0-P was noted for possible consideration in the case of a sterol with unexpectedly high retention time. Although 18:0-P to 20:0-P eluted prior to the sterols, these three IS solutions were left in the FAP-mix because they could be interesting in the future for the indexing of more volatile molecules in the silylated unsaponifiable matter eluting in this retention range. Possible examples feature diterpenes and tocochromanols (which eluted between 18:0-P and 23:0-P) as well as the phytosterol precursor squalene (which eluted between 18:0-P and 19:0-P). Compared to the classic use of *n*-alkanes as internal standards, e.g., in the analysis of volatiles [27], FAPs have the decisive advantage that their GC/MS spectra feature only very few abundant fragment ions (Fig. 2b), with distinct predominance of *m/z* 113 and *m/z* 126, irrespective of the chain length [28]. This was crucial for the implementation of the GC/MS-SIM mode because all FAPs in the IS mix could be monitored by only these two fragment ions, which are barely present in the GC/MS spectra of silylated sterols.

### Stability of the retention indices

FAP retention indices (RI_FAP_) of silylated sterols in the semipure β-sitosterol standard (unsaturated sterols calculated as β-sitosterol ~ 85%, along with low shares of cholesterol, campesterol, and stigmasterol) were highly reproducible (see Electronic Supplementary Material (ESM) Table S1). Fluctuations in individual measurements were generally within ∆RI_FAP_ < 1. This allowed us to present retention data of sterols by means of RI_FAP_ instead of the commonly used RRT which are known to be less stable [20].

Long-term stability of the RI_FAP_ was tested by measuring characteristic (silylated) sterols from four oils at intervals of 6 and 2 weeks (1.5 months in total, ESM Table S2). Resulting fluctuations of three sterols (clerosterol, ∆7-sitosterol, and cycloartenol) and the triterpene alcohol β-amyrin were slightly larger (> 1.5 RI_FAP_ units) than in the initial standard solution, but never > 2.1 RI_FAP_ units (ESM Table S2). These four compounds have in common that they co-eluted with another sterol or unknown compounds (valid for clerosterol) in samples. Overall, however, variations were relatively small, and all peaks could be correctly assigned by combining the RI_FAP_ value with GC/MS data. Therefore, the impact of matrix effects and co-elutions on the RI_FAP_ was considered to be negligible. However, the variations caused the listing of RI_FAP_ values by whole numbers and naming the first decimal was omitted.

### Implementation of retention time locking

Long-term measurements (almost 1 year) indicated that RI_FAP_ values and separation efficacy slightly changed with the aging of the GC column (ESM Fig. S1 and Fig. S2). Moreover, cutting the GC column as part of routine system maintenance affected the stability of RI_FAP_ values. Namely, shortening the GC column by ~ 1.5 m not only decreased retention times but also unevenly affected RI_FAP_ values (ESM Fig. S1, circled). For instance, RI_FAP_ values partly dropped or increased (e.g., in the range of ∆5-campesterol to ∆7-campesterol) and later on dropped again (e.g., stigmasterol, ESM Fig. S1c). In order to omit frequent substitution of the column, retention time locking was introduced by locking the retention time of 24:0-P at 32.972 min. Subsequent measurements every 2 weeks with and without retention time locking (RTL) showed that relocking after routine system maintenance was sufficient for high long-term quality of RI_FAP_ values. However, strong changes in column length (> 1 m) should be omitted in order to exclude that resulting retention time values get outside the calibration range.

**Fig. 4:**
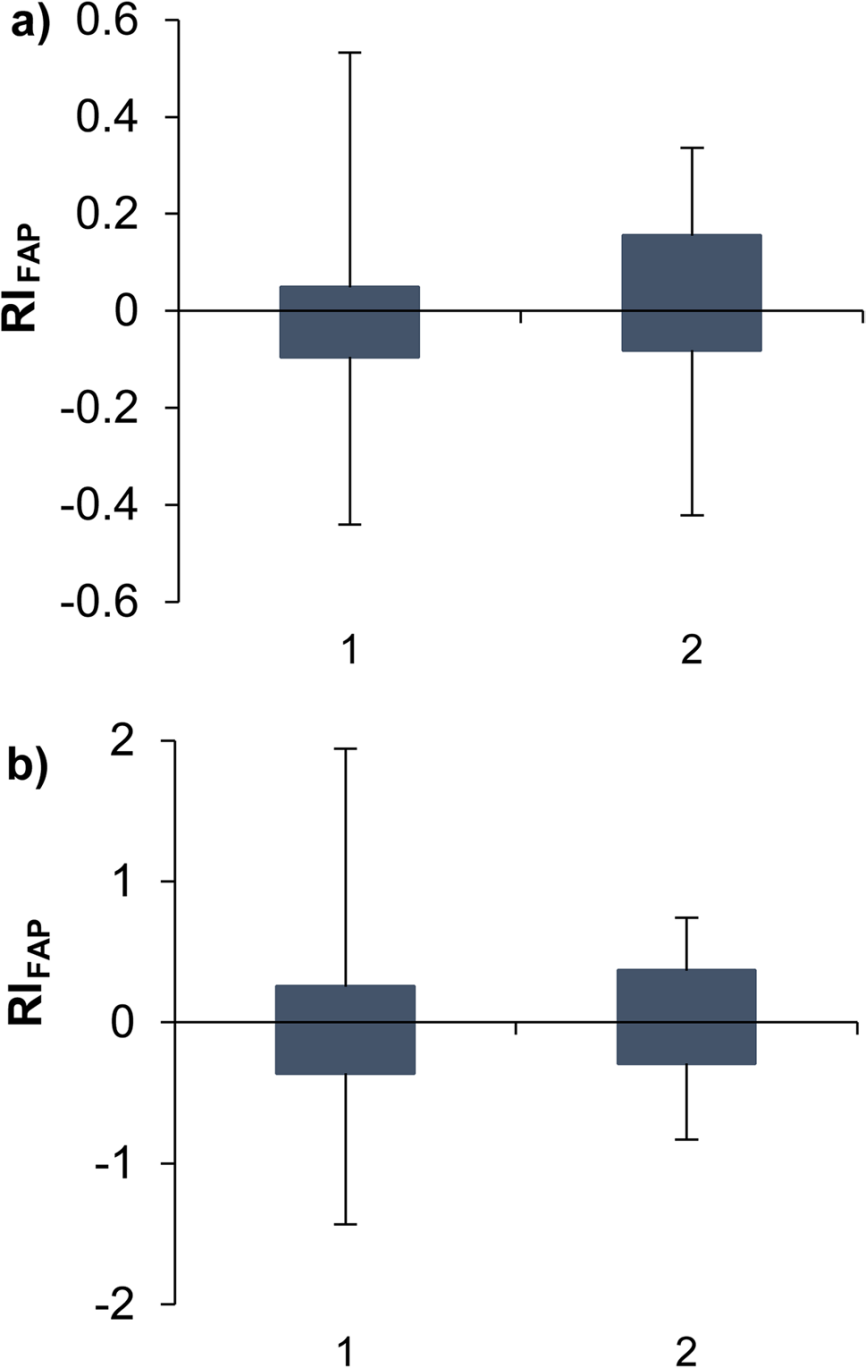
Box-plots of the shifts in the RI_FAP_ determined for silylated (a) cholesterol and stigmasterol (b), determined (1) with a non-locked and (2) with a retention time locked (RTL) GC/MS-SIM method in the course of 5 months. Outliers are excluded

All in all, RTL considerably improved the stability of RI_FAP_ values of individual silylated sterols compared to the non-locked procedure (Fig. 4, ESM Fig. S3) Stronger deviations were observed when peak tailing occurred with FAP-IS (mid-November and early January) (ESM Fig. S3, circled, and Fig. S4). Subsequent system maintenance (replacing guard column and glass liner) resulted again in correct RI_FAP_ values (ESM Fig. S3). Hence, routine measurements of the FAP-IS mix and inspection of peak shapes were introduced for quality control. Overall, the implementation of RTL improved the performance of ~ 90% of all sterols.

### Selection of suitable GC/MS-SIM ions for silylated sterols

The most relevant structural information of silylated sterols is provided by the molecular ion. Sterols feature 27–31 C-atoms and 0–3 DBs, which adds up to 5 × 4 = 20 molecular ions (Table 2). Differentiation of isomers required the additional monitoring of diagnostic fragment ions (and RI_FAP_ values). In addition, two characteristic fragment ions for FAPs (*m/z* 113 and 126) had to be included in the method. Previous investigations showed that 15–18 SIM values can be measured simultaneously without loss of quality [29, 30]. In the present case, 17 SIM ions were selected and still provided a cycle per second rate of > 2. Hence, time windows were implemented/required in order to keep information high (Tables 1 and 2).

**Table 2:** Selected molecular and fragment ions of the silylated sterols and triterpene alcohols (mass defect of the isotopes is included)

		27	28	29	30		31
Molecular ion	0	460.4	474.4	488.4	502.5		516.5
	1	458.4	472.4	486.4	500.4		514.5
	2	456.4	470.4	484.4	498.4		512.5
	3	454.4	468.4	482.4	496.4		510.5
		Saturated	∆5-Sterol	∆7-Sterol	∆5,7- or ∆5,8-sterols	∆24-Sterols	Amyrins
Fragment ion		215.2	129.1, 213.2, 255.2	255.2, 213.2	253.2, 211.2	296.2	189.2, 218.3

With regard to fragment ions, the following ones were considered most important for silylated sterols. Stanols show the base peak at *m/z* 215, which is formed by loss of the entire side chain along with the three carbons C-15 to C-17 of ring D and the TMSOH group [31]. ∆5- and ∆7-sterols both feature the diagnostic *m/z* 213 and *m/z* 255; the latter results from elimination of the entire side chain on C-17 along with the TMSOH group. Both groups can be distinguished by *m/z* 129, which is the base peak of ∆5-sterols (TMSOH group and carbons C-1 to C-3 of ring A, Fig. 1). Moreover, sterols with two DBs in ring B (∆5,7-/∆5,8-sterols) feature the corresponding fragment ions at *m/z* 211 and *m/z* 253. Lastly, *m/z* 296 ([M-84-TMSOH]^+^) was included in the method, because it verifies ∆5-sterols with a second DB on C-24(C-24^1^) or C-24(C-25).

Finally, two fragment ions (*m/z* 218 and *m/z* 189) were selected because they allowed for indicating pentacyclic triterpene alcohols, which are also partly abundant in the unsaponifiable matter of plants and which are commonly listed as 4,4-dimethylsterols [13–15]. GC/MS spectra of pentacyclic triterpene alcohols usually show a weak molecular ion (ESM Fig. S5), but with abundant fragment ions at *m/z* 218 and *m/z* 189. Both ions are formed by a retro-Diels–Alder reaction in ring C [32, 33]. Moreover, the abundance ratio of both fragment ions allowed distinguishing amyrins and further pentacyclic triterpene alcohols with a DB on C-12 (higher abundance of *m/z* 218) from those with a DB on C-13 and lupeol (higher abundance of *m/z* 189). Further diagnostic fragment ions such as *m/z* 343 for spinasterol [11] may be helpful as well, but were not included in the final method in order to keep track on basic features. However, this and further SIM values [9] may be selectively measured in separate confirmatory GC/MS runs.

Parallel measurement of eight molecular ions allowed for the determination of nine fragment ions and two SIM values for IS in order cover the full array. Hence, time windows had to be implemented. The time windows had to be set very tightly (four time windows within 10 min). In order to find appropriate positions without loss of information, each sample was initially analyzed by two independent GC/MS-SIM runs on the 20 molecular ions. Namely, molecular ions of C_28_- and C_29_-sterols were measured in SIM method 1 and those of C_27_-, C_30_-, and C_31_-sterols in SIM method 2 (Table 1). An overlap of C_27_- and C_31_-sterols could be ruled out, as GC retention times of sterol-TMS are increasing with the number of carbon atoms [34]. Consequently, the molecular ions of C_27_-sterols were only measured in the first part of the GC run (here: until 29.6 min), and C_31_-sterols thereafter until the end of the run. Most fragment ions were measured throughout the run, with the exception of pentacyclic triterpene alcohols, because these C_30_-compounds eluted within (in the middle section) SIM method 2 [15, 35].

### GC/MS-SIM analysis of the unsaponifiable matter of four edible oils on silylated sterols

Thirty sterols and triterpene alcohols were detected in the silylated unsaponifiable matter of four different oils by GC/MS-SIM (Table 3). Most of them could be verified by GC/MS in full scan mode, but some low abundant sterols could only be detected via GC/MS-SIM. Sterols were detected with 27–31 C-atoms and 0–2 DBs in different ∆-positions. The highest structural diversity was observed in the group of C_29_-sterols (*n* = 12), which included the most prominent sterol, β-sitosterol. Varieties of C_28_- (*n* = 7) and C_30_-sterols (*n* = 8, including three triterpene alcohols) were also high while only one C_27_-sterol (cholesterol, #1, 1 DB) and two C_31_-sterols were detected.

**Table 3:** GC/MS data along with assignment level and semi-quantitative data* of 30 silylated sterols to a sample of rapeseed, hemp, corn, and sunflower oils

	Sterol	RRT**	RI_FAP_	cn:DB	DB-∆position	M^+^	Base peak	Assignment level	Rapeseed oil	Hemp oil	Corn oil	Sunflower oil	Source
ISTD	5α-Cholestane	1.00	1868	S27:0	0	372		1					
#1	Cholesterol	1.29	2129	S27:1	∆5	458	129	1	0.06	0.2	0.02		
#2	Brassicasterol	1.34	2169	S28:2	∆5. 22	470	129	2	5.5				
#3	24-Methylenecholesterol	1.39	2217	S28:2	∆5. 24	470	129	2	0.2	0.05	0.1		
#4	Campesterol	1.40	2225	S28:1	∆5	472	129	1	34.9	8.6	13.3	2.6	
#5	Unknown 1***	1.41	2230	S29:2	∆n/a	484	470	4				0.1	
#6	Campestanol	1.41	2235	S28:0	0	474	215	2	0.1	0.06	0.6		
#7	Unknown 2 (∆8-campesterol)^a^***	1.42	2247	S28:1	∆8	472	472	3	0.1				[41]
#8	Stigmasterol	1.42	2247	S29:2	∆5. 22	484	129	1		1.0	5.0	4.2	
#9	Unknown 3***	1.45	2274	S29:1	∆n/a	486	129	4	0.2	0.05	0.05	0.05	
#10	Unknown 4***	1.45	2274	S28:2	∆n/a	470	129	4					
#11	∆7-Campesterol	1.45	2284	S28:1	∆7	472	255/472	2	0.07	0.05	0.02	0.8	[15]
#12	Clerosterol	1.46	2291	S29:2	∆5, 25	484	129	2	0.08	0.08	0.1	0.3	[15]
#13	Lanosterol***	1.48	2306	S30:2	∆8, 24	498	129	2		78.4		64.4	
#14	β-Sitosterol	1.48	2306	S29:1	∆5	486	129	1	57.0		74.2		
#15	Sitostanol	1.49	2319	S29:0	0	488	215	2		7.2	5.8		
#16	∆5-Avenasterol	1.49	2320	S29:2	∆5, 24	484	129	2	1.6			2.8	
#17	β-Amyrin	1.50	2327	S_t_30:2	∆12 ^6^*r*_17,18_	498	218 (129)	2	tr	1.9	tr	2.2	
#18	Unknown 5 (∆8-sitosterol)^a^***	1.50	2329	S29:1	∆n/a	486	486	3					[41]
#19	Unknown 6 (butyrospermol)^a^	1.51	2336	S30:2	∆7, 24	498	129	3		0.5			[42]
#20	Stigmasta-5,24(25)-dienol	1.51	2342	S29:2	∆5, 24 (25)	484	129	2	0.2	0.3	0.2	0.4	[15]
#21	Unknown 7***	1.53	2354	S31:2	∆n/a	512	129	4				0.5	
#22	Gramisterol	1.53	2354	S29:2	∆7, 24 (24^1^)	484	129	2		0.2	0.1		[31]
#23	∆7-Sitosterol	1.54	2364	S29:1	∆7	486	255	2	0.09	0.6	0.2	16.3	
#24	α-Amyrin	1.54	2365	S_t_30:2	∆12 ^6^*r*_17,18_	498	218	2					
#25	Cycloartenol	1.54	2371	S30:2	∆24 ^3^*r*_9,10_	498	129	2	0.07	0.5	0.2	1.9	
#26	Lupeol	1.54	2371	S_t_30:2	∆20 ^5^r_17,18_	498	189	2					
#27	∆7-Avenasterol	1.55	2378	S29:2	∆7, 24 (24^1^)	484	253	2		0.1	0.07	1.5	
#28	24-Methylenecycloartanol	1.60	2423	S31:2	∆24 ^3^*r*_9,10_	512	129	2		0.04	0.1	0.4	
#29	Unknown 8***	1.63	2442	S30:1	∆n/a	500	500	4				0.2	
#30	Citrostadienol	1.64	2457	S30:2	∆7, 24	498	129	2		0.1	0.04	1.4	

*RRT*, retention time relative to the internal standard 5α-cholestane; *RI*_*FAP*_, FAP retention index; *cn*, carbon number; *DB*, number of double bonds with S indicating sterols and stanols and *S*_t_ indicating triterpenol alcohols (e.g., amyrins); *∆*, location of double bond(s); *n/a*, not available; ^*x*^*r*_*y*_, ring system; *x*, number of carbon atoms in the ring; *y*, place in the sterol backbone where the ring is added; *M*^+^, trimethylsilylated molecular ion used for GC/MS in selected ion monitoring (SIM) mode. Assignment levels: (1) standard available; (2) major sterol, verified by GC/MS data; (3) tentative assignment, (4) unknown: GC/MS equivocal or of insufficient quality

^a^Most likely proposed structure

^*^The contribution in percentage was derived from the total ion current of the GC/MS-SIM method. Due to the lack of reference standards and the partly very low concentrations in the solutions, it is currently impossible to determine if all compounds produce the same response at the same concentration. Hence, the presented data should not be mixed with the quantitative composition. Instead, the data gives a good impression on the relevance of the individual silylated sterols in the GC/MS-SIM chromatograms

^**^RRT: 18:0-P (0.93), 19:0-P (1.03); 20:0-P (1.14); 21:0-P (1.26); 22:0-P (1.38); 23:0-P (1.47); 24:0-P (1.57); 25:0-P (1.70); 26:0-P (value 1.85 determined in initial measurements, not included in the mix)

^***^Minor sterols which were not described before in the corresponding edible oil

The most important mass spectral data (base peak, molecular ion, the number of carbon atoms, and DB equivalents) of the sterols and triterpene alcohols are summarized in Table 3. Since DBs and additional rings (here: cyclopropane rings) are isobaric, DB numbers were only assigned when unequivocally known while sterols with a cyclopropane ring (e.g., 24-methylenecycloartanol) were listed in ^*x*^*r*_*y*_ mode, where *x* denotes the number of C-atoms of the ring and the *y* denotes the connection points on the sterol backbone (e.g., ^3^*r*_9,10_ for 24-methylenecycloartanol, Table 3). Similarly, pentacyclic triterpene alcohols like amyrins were marked with the same ^*x*^*r*_*y*_ system (e.g., ^6^*r*_17,18_ for α-amyrin, Table 3).

Detected sterols were categorized by using a 4-level system. Level 1 was assigned to sterols verified by means of an authentic reference standard, whereas level 2 was assigned to major sterols which could be unequivocally verified by GC/MS and literature data. Together, levels 1 and 2 could be assigned to 22 sterols (Table 3). Level 3 was used to specify three sterols which could be tentatively assigned while level 4 was used when abundance was too low to collect structural information (Table 3). This is applied to the remaining five sterols. Seven of the level 3 and 4 sterols (all except butyrospermol) and lanosterol (Table 3, *) have not been described in previous studies of these four oils [36–44], because of their low concentration and the additional co-elution of lanosterol with β-sitosterol.

#### C_28_-sterols

The most relevant representative of this group was campesterol (#4, 1 DB), which was the second most abundant sterol in all but sunflower oil. Campesterol (#4) is commonly found in vegetable oils [36, 37, 44]. Rapeseed oil featured brassicasterol (#2, 2 DBs), which is characteristic for *Cruciferae* [41]. Other C_28_-sterols frequently found in edible oils were 24-methylenecholesterol (#3, 2 DBs) and campestanol (#6, no DB) [39, 45]. These were detected in all oils except for sunflower oil.

In addition, ∆8-campesterol (#7, or its epimer ergost-8-enol) was tentatively identified (level 3) in rapeseed oil due to the molecular ion at *m/z* 472 (C_28_-sterol, 1 DB) and its reported elution between ∆5- and ∆7-campesterol [25, 26]. More details could not be obtained from the GC/MS spectrum because the peak co-eluted with the more abundant stigmasterol (C_29_-sterol, 2 DBs). Sterol 4 (#10, 2 DBs, level 4) was very low abundant in all samples and it co-eluted with the unknown C_29_-sterol 3 (#9, level 4), which was also detected in all four oils.

#### C_29_-sterols

The twelve C_29_-sterols featured no (*n* = 1), one (*n* = 4), or two DBs (*n* = 7) (Table 3). β-Sitosterol (#14, 1 DB) dominated in all screened oils which is in good accordance with literature data [36, 37, 44]. At slightly higher RI_FAP_ value, ∆5-avenasterol (#16, 2 DBs) co-eluted in two oils with sitostanol (#15, no DB), but could be distinguished by GC/MS-SIM due to both having different molecular ions and fragment ions.

Other common C_29_-sterols (level 1 or 2) in the samples were stigmasterol (#8, 2 DBs); clerosterol (#12, 2 DBs); ∆7-avenasterol (#27, 2 DBs); stigmasta-5,24(25)-dienol (#20, 2 DBs); and ∆7-sitosterol (#23, 1 DB). ∆7-Sitosterol is the ∆7-isomer of β-sitosterol, whereas the other sterols were isomers of ∆5-avenasterol, with one DB at ∆5- or ∆7-position and the second one located in the side chain. ∆7-Avenasterol was detected in all oils except in rapeseed oil, whereas the other four sterols were detected in all samples. Gramisterol (#22, 2 DBs, level 2), which co-eluted with sterol 7 (#21, 2 DBs, level 4), particularly stood out, as it was the only detected 4-methylsterol with less than 30 carbon atoms. It was detected in three out of the four analyzed oils and has already been described in sunflower oil and corn oil by Schwartz et al. [36]. ∆8-sitosterol (#18, 1 DB, level 3) was tentatively identified in hemp oil and sunflower oil analogous to ∆8-campesterol (see previous section). Indeed, it co-eluted with β-amyrin (#17, 2 DBs), but due to the different fragmentation of amyrins, both compounds could be differentiated by the GC/MS-SIM method.

Unknown sterol 1 (#5, 2 DBs) was detected in traces in sunflower oil. It eluted between campesterol (#4, 1 DB) and campestanol (#6, no DB), with the latter one being absent in sunflower oil. The last detected C_29_-sterol (sterol 3, #9, 2 DBs, level 4) was already mentioned in the previous section, as it co-eluted with unknown sterol 4 (#10, level 4) and thus the fragmentation pattern could not be studied in detail.

#### C_30_- and C_31_-sterols

All sterols of these two groups were either 4-methyl- or 4,4,-dimethylsterols, or belonged to the pentacyclic triterpene alcohols. Due to the sensitive detection of the latter group by means of *m/z* 189 and *m/z* 218, β-amyrin (#17, 1 DB, ∆12, ^6^*r*_17,18_) could be detected in all samples and α-amyrin (#24, 1 DB, ∆12, ^6^*r*_17,18_) was also detected in three samples.

The third pentacyclic triterpene alcohol, lupeol (#26, 1 DB, ∆20, ^5^*r*_17,18_), was difficult to differentiate from the co-eluting cycloartenol (#25, 1 DB, ^3^*r*_9,10_) since both GC/MS spectra featured the characteristic fragment ion at *m/z* 189. Consequently, assignment of lupeol additionally required the presence of *m/z* 218 (see above), which was observed in sunflower oil, whereas cycloartenol was detected (fragment ions *m/z* 215 and *m/z* 255) in all samples.

24-Methylenecycloartanol (#28, 1 DB, ^3^*r*_9,10_) and citrostadienol (#30, 1 DB) were found in all oils but rapeseed oil, whereas traces of the 4,4-dimethylsterol lanosterol (#13, 2 DBs) were detected only in hemp and sunflower oil in larger quantities. Partial co-elution with the dominant β-sitosterol and comparably low abundant SIM ions hampered the good detection of lanosterol. In the presence of a 14-methyl substituent, diagnostic fragment ions are weakened until completely hindered [31]. The only detectable diagnostic ion for lanosterol apart from the molecular ion was *m/z* 215 (ESM Fig. S6), which was also low abundant in the GC/MS spectrum. As this fragment ion was overlaid by the fragment ions of β-sitosterol (forms mainly *m/z* 213), lanosterol could only be identified by its molecular ion. 4-Monomethylsterols like citrostadienol formed the same fragment pattern in GC/MS-SIM mode (ESM Fig. S6), because diagnostic fragment ions in the ring system are shifted by 14 u (e.g., *m/z* 213 ➔ *m/z* 227) [31].

Butyrospermol (#19, 2 DBs, level 3) was tentatively identified in hemp oil by comparison with previously published GC/MS data [38, 43, 46]. Sterol 7 (#21, level 4) showed the molecular ion at *m/z* 512 which indicated the presence of 32 carbon atoms and 2 DBs. Because of its low abundance in sunflower oil and exactly the same RI_FAP_ (full co-elution, Table 3) with gramisterol (#22, C_29_, 2 DBs), structural details could not be established. However, the benefits of the GC/MS-SIM methods were apparent because both sterols could be distinguished from each other. Similarly, the unknown sterol 8 (#29, 1 DB, level 4) was detected in traces in sunflower oil. It eluted between 24-methylenecycloartanol (#28) and citrostadienol (#30) from the GC column.

### Establishing of the time windows

**Fig. 5:**
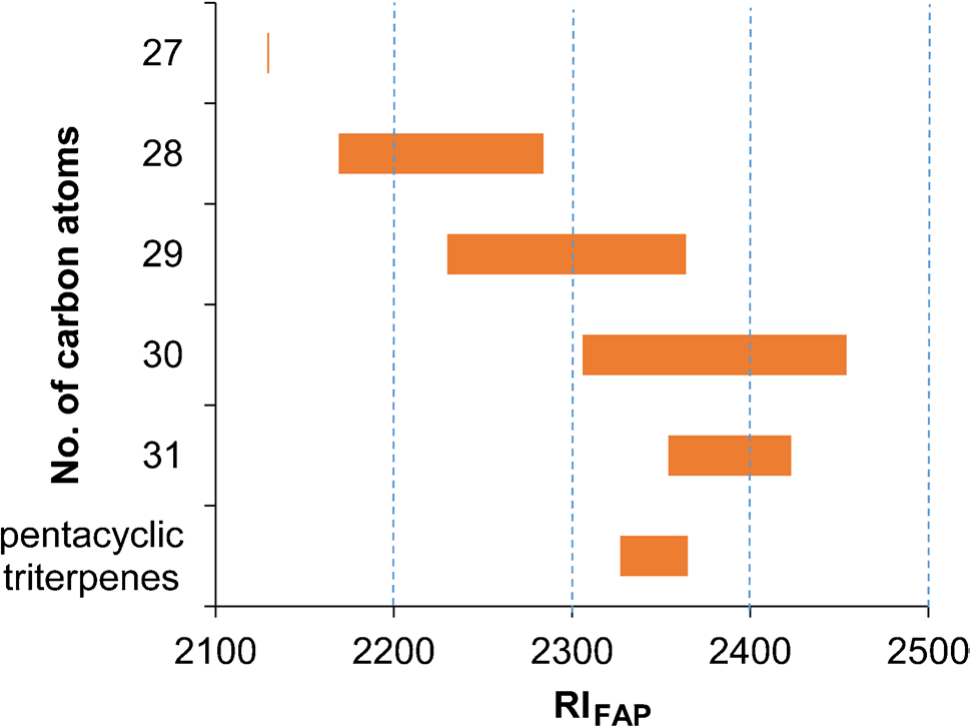
Retention range (RI_FAP_) of the 30 silylated sterols and pentacyclic triterpenes grouped after their number of carbon atoms on an Optima 5 HT (30 m, 0.25 mm i.d., 0.25-μm film)

In the last step, the two initial GC/MS-SIM methods were merged by establishing time windows based on the first and last eluting sterol of a given chain length (Fig. 5). Although all groups were overlapping (except for cholesterol, which was the only detected C_27_-sterol), this could be managed by means of time windows with eight molecular ions each (two chain lengths) with one exception. Namely, the unknown C_31_-sterol (#21) eluted before the last C_29_-sterol (#27, Δ7-avenasterol). Hence, the molecular ion of sterol #21 (*m/z* 512) was included in time window 3 by substitution of *m/z* 496.4 because C_30_-sterols with 3 DBs were not detected at all (Table 4).

**Table 4:** GC/MS-SIM parameters of the final determination method of silylated sterols in edible oils

Time window	RI_FAP_	Retention-independent ions	Group	Retention-dependent ions
1	2100–2200	113.0; 126.0; 129.1; 211.2; 213.2; 215.2; 253.2; 255.2; 296.2	C_27_	454.4, 456.4, 458.4, 460.4
			C_28_	468.4, 470.4, 472.4, 474.4
2	2200–2300		C_28_	470.4, 472.4, 474.4
			C_29_	482.4, 484.4, 486.4
3	2300–2400	113.0; 129.1; 211.2; 213.2; 215.2; 253.2; 255.2; 296.2	C_29_	482.4, 484.4, 486.4, 488.4
			C_30_	498.4, 500.4
			C_31_	512.5
			Amyrins	189.2, 218.3
4	2400–2500	113.0; 126.0; 129.1; 211.2; 213.2; 215.2; 253.2; 255.2; 296.2	C_30_	496.4, 498.4, 500.4, 502.5
			C_31_	510.5, 512.5, 514.5, 516.5

Since the time windows had to be set very tightly, RTL played a particular role in order to avoid shifts and thus loss of compounds (Table 4). At this point, it should be noted that the present GC/MS-SIM method was based on 30 sterols. Further oils will likely include other (unknown) sterols [31]. Hence, analysis of additional edible oils may necessitate slight shifts in the time windows. However, the [M-14]^+^ signals need to be carefully examined as well (see “Materials and methods”). As 14-methylsterols like 24-methylenecycloartanol (#28, 1 DB, ^3^*r*_9,10_ M^+^ at *m/z* 512) form a prominent [M-15]^+^ ion (*m/z* 497), the respective ion emerging from the ^13^C isotope peak (nominally [M-14]^+^) will be displayed in the corresponding SIM window more intense than M^+^. The presence of another sterol with one carbon atom less (isobaric with [M-14]^+^) needs to be excluded by comparison of the relative intensities of these ions. This is easy to be carried out in the case of known sterols but challenging in the case of unknown sterols. Hence, a permanent inspection of the setup is mandatory, especially in the initial phase. Here, it could be advisable to occasionally run the initial two SIM runs (Table 1).

## Conclusions

FAP retention indices were successfully introduced for the analysis of sterols. Implementation of a RTL method improved the precision of RI_FAP_ values. The resulting RI_FAP_ values proved to be a good alternative to the conventionally used RRT to internal standards like 5α-cholestane or betulin. Application of the GC/MS-SIM mode enabled the detection of thirty sterols and triterpene alcohols in only four different edible oils.

## Supplementary Information

Below is the link to the electronic supplementary material.

Supplementary file1 (PDF 222 KB)
