# Supplementary material for: GC/EI-MS method for the determination of phytosterols in vegetable oils
Source: Anal Bioanal Chem. 2021 Oct 30;414(2):1061–71. doi: 10.1007/s00216-021-03730-9 (PMC8724214; doi:10.1007/s00216-021-03730-9)
Supplement: Supplementary file 1 — Supplementary file1 (PDF 222 KB) [file 216_2021_3730_MOESM1_ESM.pdf]

Analytical and Bioanalytical Chemistry

Electronic Supplementary Material

GC/EI-MS Method for the Determination of Phytosterols in Vegetable Oils

Sarah Schlag[1], Yining Huang[1], Walter Vetter[1]*

[1]University of Hohenheim, Institute of Food Chemistry (170b), Garbenstraße 28, D-70599 Stuttgart, Germany

*Corresponding author

Tel: +49 711 459 24016

Fax: +49 711 459 24377

E-mail: walter.vetter@uni-hohenheim.de

Table S1 FAP retention indices (RI_{FAP}) of silylated sterols in a crude β-sitosterol standard (unsaturated sterols calculated as β-sitosterol ~85%) as determined by GC/MS-SIM

injected amount [ng]	measurement	cholesterol	campesterol	stigmasterol	β-sitosterol
12.45	1	2127.7	2224.7	2246.5	2306.0
	2	2127.6	2224.6	2246.5	2305.9
24.9	1	2127.8	2224.9	2246.7	2305.9
	2	2127.5	2224.8	2246.5	2305.8
49.8	1	2127.8	2224.5	2246.7	2306.0
	2	2127.6	2225.0	2246.8	2306.0
74.7	1	2127.8	2224.7	2246.7	2306.2
	2	2127.7	2224.9	2246.9	2306.1
99.6	1	2127.9	2224.8	2246.8	2306.4
	2	2127.6	2224.9	2246.6	2306.4
124.5	1	2128.0	2224.9	2246.6	2306.3
	2	2127.7	2224.7	2246.9	2306.4
maximum		2128.0	2225.0	2246.9	2306.4
minimum		2127.5	2224.5	2246.5	2305.8
deviation		0.5	0.5	0.4	0.6
STABW		0.1	0.1	0.1	0.2

Table S2 Comparison of FAP retention indices (RI_{FAP}) of silylated sterols and triterpene alcohols as determined by GC/MS-SIM in samples from different series of measurements

	A	B	C	D	max	min	max. dev.	STDEV
24-methylenecholesterol	-	2215.3	2216.0	2215.6	2216.0	2215.3	0.7	0.4
campesterol	2224.4	2223.1	2223.7	2223.7	2224.4	2223.1	1.3	0.5
stigmasterol	2247.2	2246.2	2246.2	2246.1	2247.2	2246.1	1.1	0.5
Δ7-campesterol	2283.5	2282.0	2282.7	2282.1	2283.5	2282.0	1.5	0.7
clerosterol	2291.2	2289.7	2289.1	2289.5	2291.2	2289.1	2.1	1.0
β-sitosterol	2306.2	2305.8	2305.6	2304.8	2306.2	2304.8	1.4	0.6
Δ5-avenasterol	2319.6	2318.7	2318.7	2318.4	2319.6	2318.4	1.2	0.5
β-amyrin	2327.3	2325.5	2326.2	2325.8	2327.3	2325.5	1.8	0.8
stigmasta-5,24(25)-dienol	2341.4	2341.0	2340.8	2340.0	2341.4	2340.0	1.4	0.6
gramisterol	2354.0	2352.9	2353.5	-	2354.0	2352.9	1.1	0.6
Δ7-sitosterol	2364.0	2363.3	2363.1	2362.0	2364.0	2362.0	2.0	0.8
α-amyrin	2365.5	-	2365.0	2364.7	2365.5	2364.7	0.8	0.4
cycloartenol	2370.9	2369.8	2369.1	2369.0	2370.9	2369.0	1.9	0.9
Δ7-avenasterol	2378.6	2377.6	2377.7	-	2378.6	2377.6	1.0	0.6
24-methylenecycloartanol	2423.2	2422.3	2422.4	-	2423.2	2422.3	0.9	0.5
citrostadienol	2457.2	2456.4	2456.8	-	2457.2	2456.4	0.8	0.4

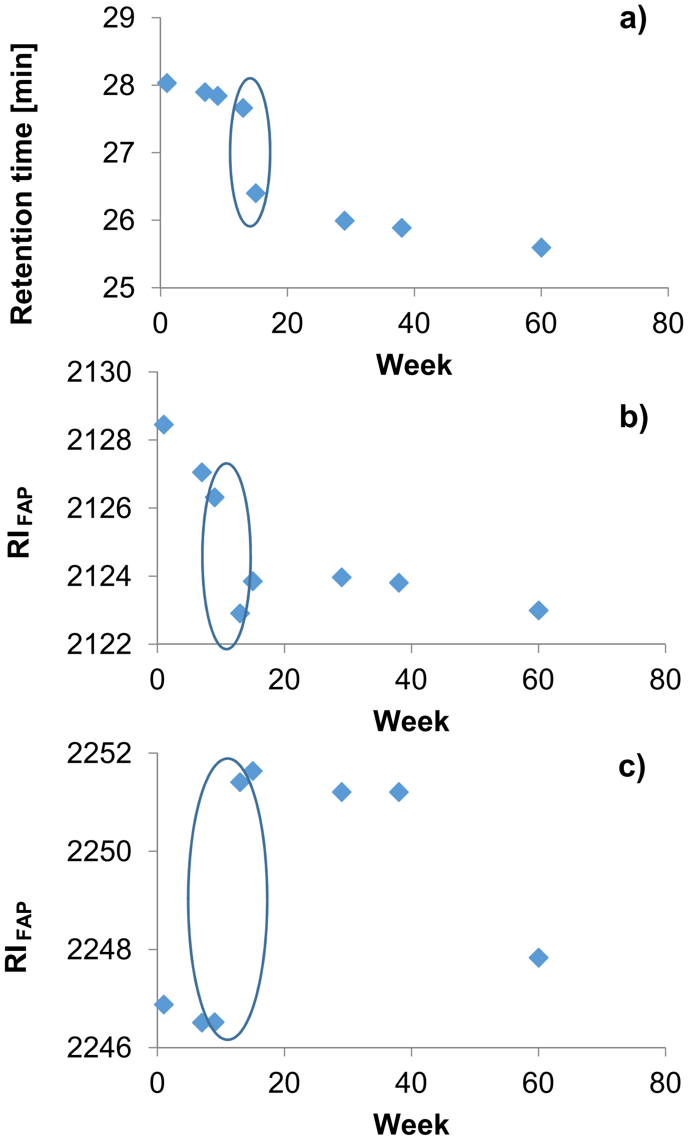

Fig. S1 Changes in the (**a**) retention time of 21:0-P, (**b**) RI_{FAP} of silylated cholesterol and (**c**) RI_{FAP} of silylated stigmasterol on the same column in the course of a year. Jumps are marked with a circle

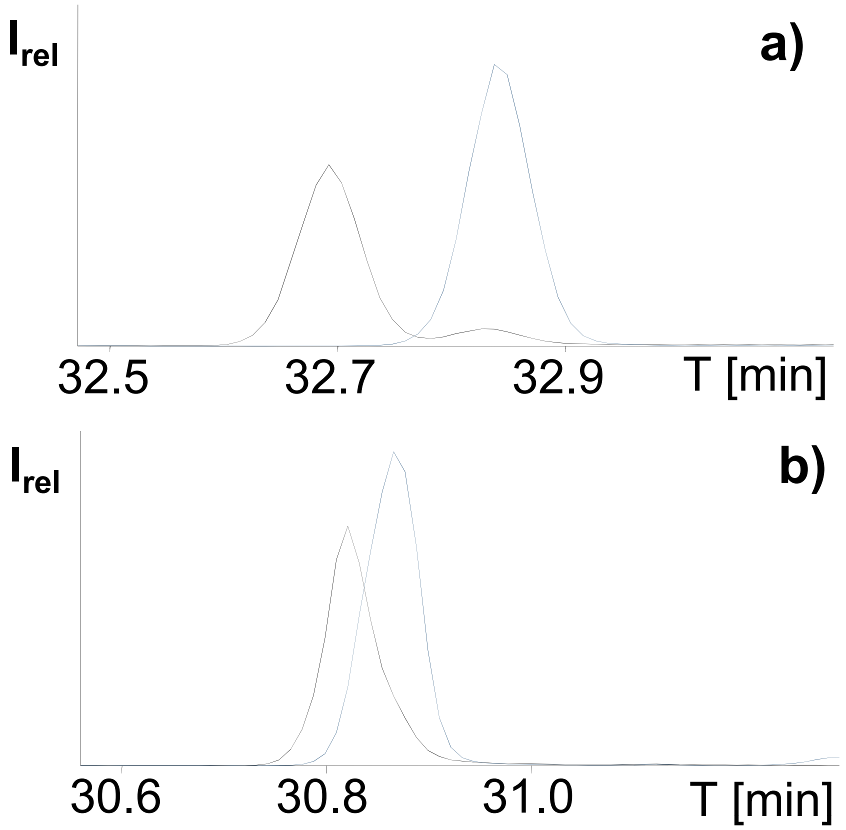

Fig. S2. GC/EI-MS SIM chromatogram of the silylated unsaponifiable matter from sunflower oil (**a**) from December 19 and (**b**) from October 20 with *m/z* 486 (blue) for β-sitosterol and *m/z* 113 (black) for 23:0-P

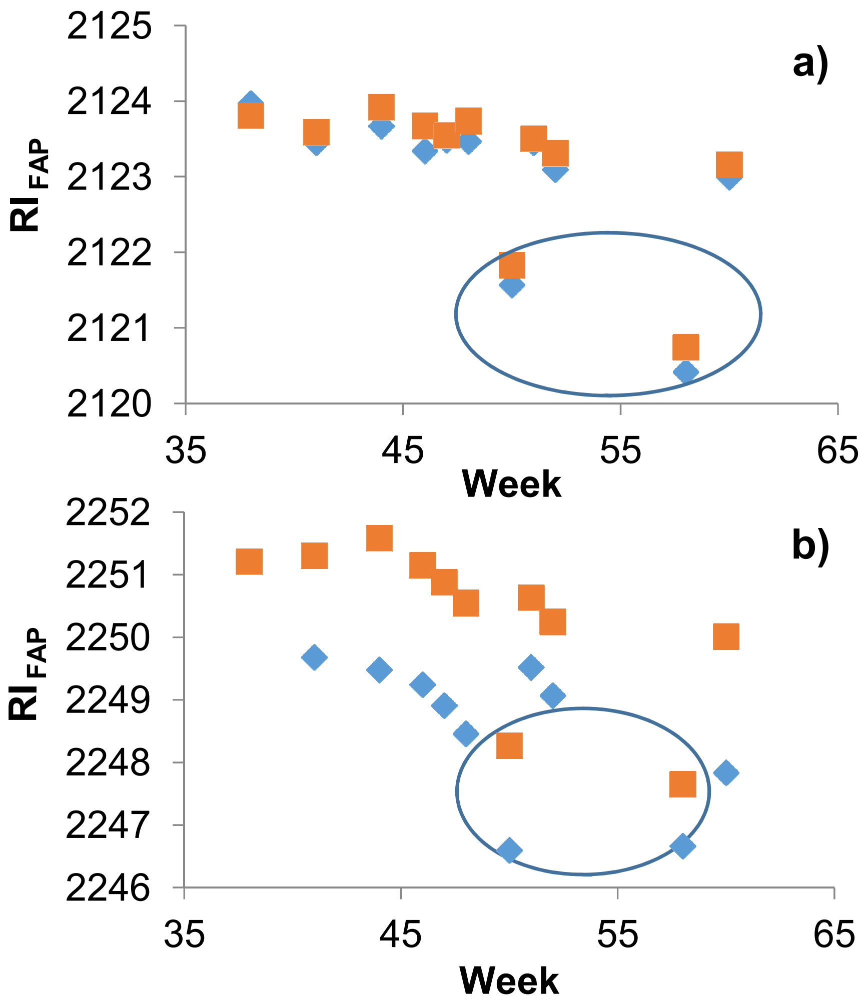

Fig. S3. RI_{FAP} from silylated (**a**) cholesterol and (**b**) stigmasterol determined with the RTL method (red, squares) and with the n-method (blue, ruts). Outliers are circled

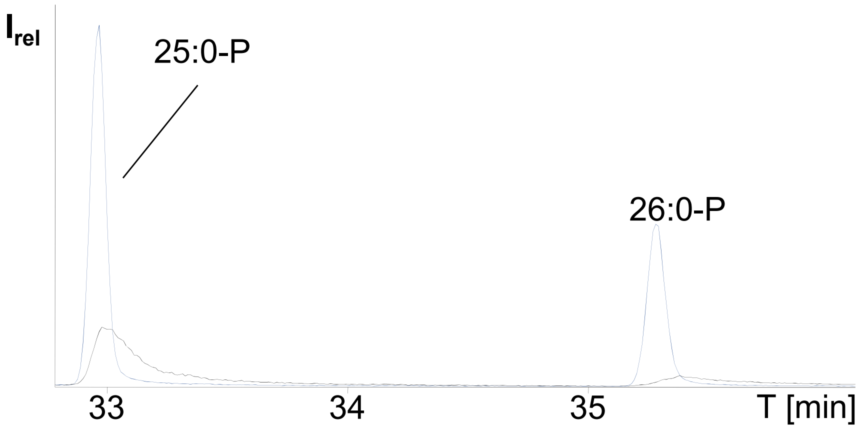

Fig. S4 GC/MS-SIM chromatogram of the FAP-IS at *m/z* 113 before (black) and after system maintenance (blue)

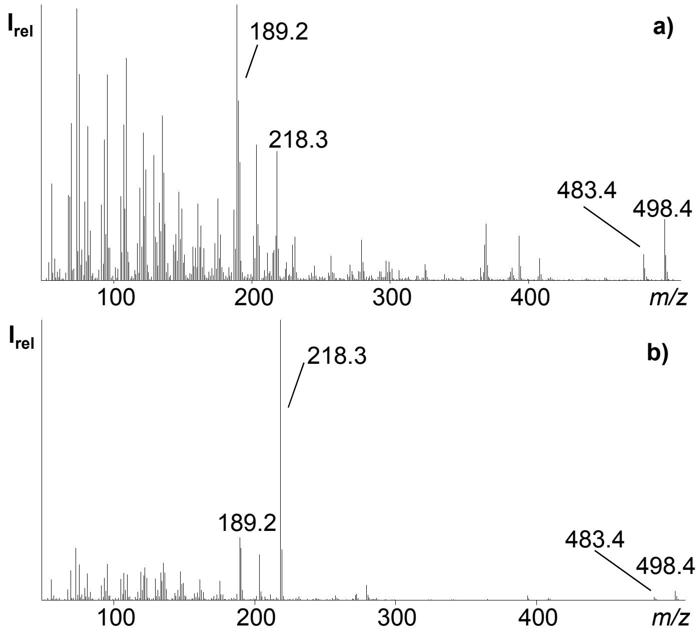

Fig. S5 GC/MS-*full scan* mass spectrums of silylated (**a**) lupeol and (**b**) α-amyrin in shea butter

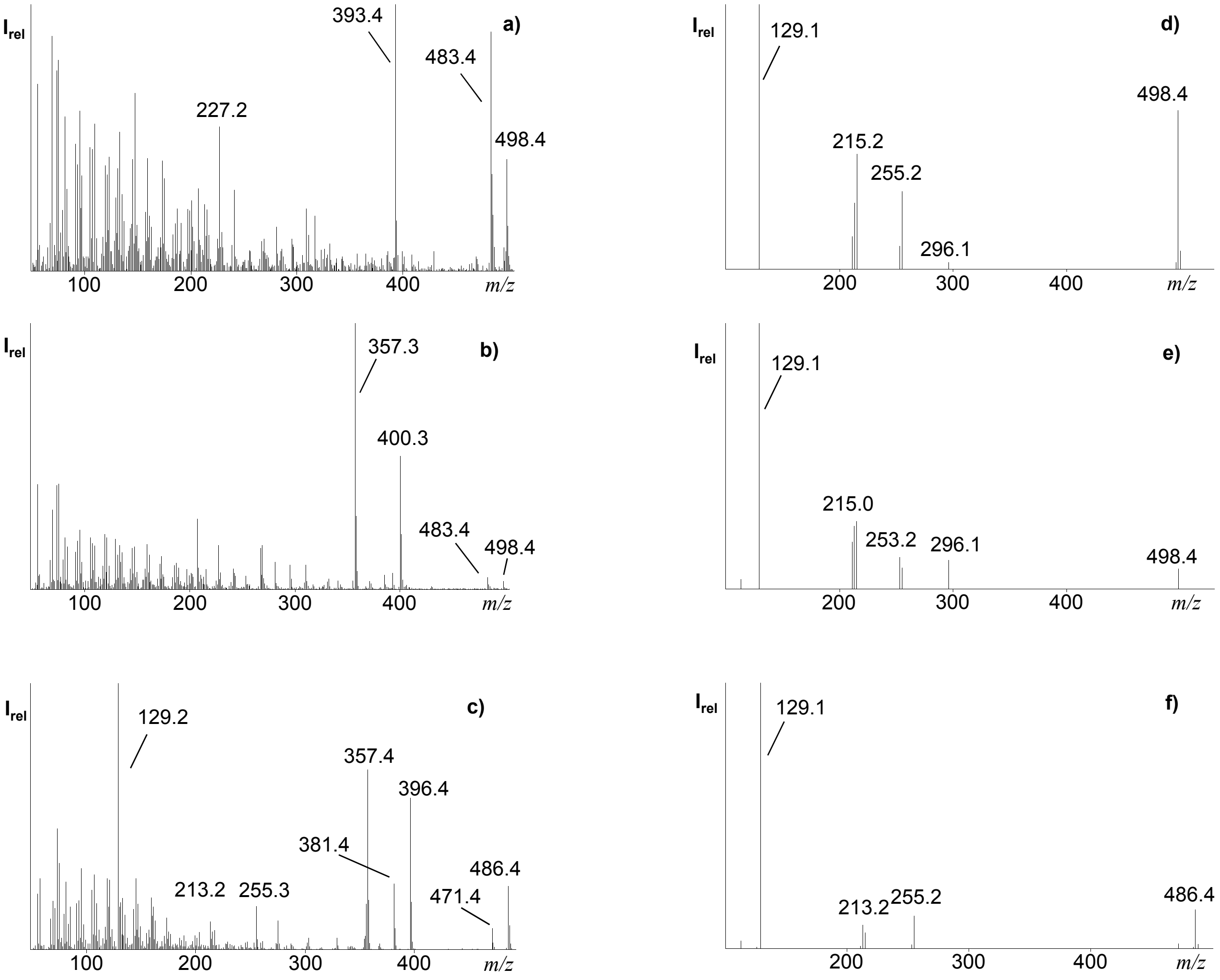

Fig. S6. GC/MS full scan mass spectra of silylated (**a**) citrostadienol, (**b**) lanosterol (measured on a Optima-5 MS (30 m, 0.25 mm i.d., 0.25 µm film thickness column) and (**c**) β-sitosterol as well as GC/MS-SIM mass spectra of silylated (**d**) citrostadienol, (**e**) lanosterol (measured on a Optima-5 MS (30 m, 0.25 mm i.d., 0.25 µm film thickness column) and (**f**) β-sitosterol
